# Lack of sexual behavior disclosure may distort STI testing outcomes

**DOI:** 10.1186/s12889-020-08768-5

**Published:** 2020-05-04

**Authors:** Navin Kumar, Laura Forastiere, Tiange Zhang, Fan Yang, Katherine T. Li, Weiming Tang, Joseph D. Tucker, Nicholas A. Christakis, Marcus Alexander

**Affiliations:** 1 Human Nature Lab, Department of Sociology, Yale University, New Haven, CT USA; 2 Department of Biostatistics, Yale School of Public Health, New Haven, CT USA; 3 University of North Carolina at Chapel Hill Project-China, No. 2 Lujing Road, Guangzhou, 510095 China; 4 Loyola University Chicago Stritch School of Medicine, Maywood, IL USA; 5 Weill Cornell Medical College, New York, NY USA; 6 Southern Medical University Dermatology Hospital, Guangzhou, China; 7 School of Medicine, University of North Carolina at Chapel Hill, Chapel Hill, North Carolina, USA; 8 Faculty of Infectious and Tropical Diseases, London School of Hygiene and Tropical Medicine, London, UK

**Keywords:** MSM, Sexual health, Sexual behavior disclosure, China

## Abstract

**Background:**

Men who have sex with men (MSM) globally have a high burden of curable sexually transmitted infections (STIs). MSM do not frequently receive rectal STI testing because of several barriers, such as not being out (disclosure of sexual behavior). We evaluate whether Chinese MSM select an STI test (rectal vs urethral) appropriate for their sexual behavior (insertive and/or receptive), and the interactions with being out.

**Methods:**

This was a secondary analysis of data from a cross sectional MSM survey conducted at a multisite randomized controlled trial (RCT) (December 2018 to January 2019) around uptake of gonorrhea and chlamydia testing among Chinese MSM (*N* = 431). We collected socio demographics, relevant medical and sexual history, and disclosure of sexual behavior (outness). We estimated the decision to test and test choice, and the extent to which disclosure plays a role in decision making.

**Results:**

Among 431 MSM, mean age was 28 years (SD = 7.10) and 65% were out to someone. MSM who indicated versatile sexual behavior and were out to someone had a 26.8% (95%CI = 6.1, 47.5) increased likelihood for selecting the rectal test vs the ure thral test, compared to those versatile and not out. Versatile MSM out to their health provider outside of the study context had a 29.4% (95%CI = 6.3, 52.6) greater likelihood for selecting the rectal STI test vs the urethral test, compared to versatile MSM not out to their health provider.

**Conclusions:**

Sexual behavior and outness may affect gonorrhea and chlamydia testing provision. Apart from clinicians, community based efforts may reduce stigma based barriers to testing.

## Background

Men who have sex with men (MSM) globally have a high burden of curable sexuall transmitted infections (STIs) [1]. The World Health Organization (WHO) estimates that there are annually 131 million and 78 million new cases of *Chlamydia trachomatis* and *Neisseria gonorrhoeae*, respectively [2]. Among MSM worldwide, gonorrhea and chlamydia are the two most common bacterial STIs [3]. The WHO recommends MSM receive regular gonorrhoea and chlamydia testing [4].

The risk of contracting STIs can vary with sexual behaviors [5]. There are a variety of ways MSM engage in intercourse, some related to preference and some not. Sometimes the reason for sexual positioning is strategic e.g. seropostioning [6, 7]. Some MSM prefer to engage in receptive anal intercourse (top), others prefer insertive anal intercourse (bottom) and some enjoy all types of intercourse (versatile) [8, 9]. A preference for receptive anal intercourse is associated with increased likelihood of a gonorrhea and chlamydia infection [5]. MSM do not frequently receive rectal STI testing because of several barriers, including: stigma, shame, fear of invasive sampling, confidentiality concerns and clinician’s time pressures [10]. Thus, both clinician and patient factors are key to rectal STI testing. While clinician factors are important, we center on patient factors because: 1) Self testing and self collection now allow rectal testing at home, prior to seeing a clinician [11, 12]. We note that self testing also happens in clinical settings [12].

Moreover, home based self testing has had several innovations, such as internet based testing which obviates the need to see a clinician [13] and social entrepreneurship models that promote self testing [14]; 2) Substantial heterogeneity in MSM preferences may drive rectal test uptake [15] along with a range of unaccounted factors such as disclosure of sexual behavior (outness); 3) The broader randomized controlled trial (RCT), from which we drew data to conduct secondary analysis of a cross sectional survey, provided a unique context where all providers were offered rectal testing, allowing us to observe differences in MSM rectal STI uptake [16]. Within these factors, the main barrier for testing is lack of disclosure [17, 18]. If MSM are unwilling to disclose their sexual be havior, the likelihood of getting tested is low [19]. We explore the relationship between outness and rectal STI testing.

The objectives of the study were to assess if MSM are more likely to select the gonorrhea and chlamydia test most representative of their sexual behavior, compared to a test less representative of their behavior; and if outness is related to the decision to select a rectal vs urethral test. Research on MSM sexual behavior does not often account for patient factors. Our study may shed light on how sexual behavior and outness may affect gonorrhea and chlamydia test provision, improving MSM STI testing efforts.

## Methods

### Study design and participants

We conducted secondary analysis of baseline data from a cross sectional survey collected through an RCT that sought to improve on STI testing rates in MSM from December 2018 January 2019 in China [20]. This RCT is henceforth referred to the parent RCT, from which we drew data to conduct secondary analysis to evaluate how outness can affect STI test uptake. The parent RCT was conducted in Guangzhou at two sites, and Beijing in a single site. All RCT sites provided free HIV testing and were administered by MSM community based organizations. Sites were selected based on MSM input, provided free HIV and syphilis testing for MSM and had capacity to deliver STI testing services during the study period. All sites were staffed with a mix of MSM volunteers, nurses, and public health staff, with no physicians. Blood draws, testing, results reporting and test follow up were handled by site based staff. Sites followed similar procedures. Our inclusion criteria was that subjects were assigned male sex at birth and identified as male, *≥* 16 years of age, reported anal intercourse with other men, did not have a gonorrhea and chlamydia test in the past year, did not previously participate in the study and were willing to provide a mobile number or WeChat ID (popular Chinese mobile application) for STI results notification. The study was approved by the Human Subjects Committee at the University of North Carolina at Chapel Hill (IRB 18–2142), Southern Medical University Dermatology Hospital (China) and Yale University. The parent RCT [20] was registered on ClinicalTrials.gov (NCT03741725). Written informed consent was obtained from all participants.

### Procedures

All testing sites offered gonorrhea and chlamydia tests to MSM waiting for free HIV and syphilis testing. After a short introduction to the gonorrhea and chlamydia test, participants decided whether to receive testing. After obtaining informed consent, we conducted patient interviews (survey instrument in supplement) from all men approached about a gonorrhea and chlamydia test, even if they declined testing. We developed the survey for study purposes. MSM were surveyed about their sexual history, STI testing history, sexual behavior and sociodemographic variables. MSM were offered gonorrhea and chlamydia tests and were given a choice to get tested either at rectal or urethral sites but not both, because of limits to free testing at the clinics. While guidelines generally suggest triple site testing (urethral, rectal, pharyngeal), [21, 22] this is not always possible in resource limited settings, such as our study. We thus provide implications generalizable to other resource-scarce settings. With MSM limited to a single test, we have the opportunity to understand the relationship between disclosure of sexual behavior and test choice. MSM were told that the urethral test was appropriate for those preferring insertive anal intercourse, while the rectal test was for those preferring receptive anal intercourse—given that gonorrhea and chlamydia infections can be site specific [23]. There was no unique choice specific to versatile behavior. MSM could select to receive both tests but would have to pay 150RMB (USD 21). Men were told that their information would be kept confidential and gonorrhea and chlamydia test results sent after a week. Program organizers updated respondents of test results through WeChat. HIV, syphilis and gonorrhea and chlamydia tests were conducted in the clinic and the results recorded. Participants with positive test results were counselled and directed to hospital resources to receive paid treatment and follow up care.

Due to resource limitations, we were not able to pay for participant treatment, but note that Chinese STI treatment is relatively affordable [24]. These tests would likely not have been done if the study had not happened, as Chinese MSM have low gonorrhea and chlamydia testing rates [15]. Our parent RCT increased gonorrhea and chlamydia testing rates and reduced cost, with the control being the community standard of care [20].

The question on disclosure was as follows:” In the past, have you told anyone about your sexuality or sexual history with men?” The following options were provided: (1)” Yes, my long term female partner/wife”; (2)” Yes, my family members”; (3)” Yes, my friends”; (4)” Yes, my healthcare providers”; (5)” No one”. Options four and five were coded as binary variables to detail sexual behavior disclosure to health providers and non**-**specific disclosure respectively. Option five captures disclosure in a non**-**specific sense i.e. anyone and is associated with improved health outcomes [25, 26]. Option four indicates disclosure to health providers, which is key to receiving appropriate healthcare [27], more so than the other group specific disclosure options. For example, men out to their healthcare provider are more likely to get HIV testing compared to those out to their family [28]. Although participants attended a specialized MSM testing clinic, this does not reflect their disclosure to their primary care or other health providers. There is significant stigma around MSM sexual behavior in China [29] and thus men may be comfortable going to an MSM centric health provider, yet not be out to their primary health provider. For example, while men were out within the context of the health clinic in the study, 35% were not out to anyone and 80% were not out to their primary health provider. Given the high rates of non**-**disclosure outside the testing clinic, we suggest137 that broader non**-**disclosure may affect in study outcomes.

### Statistical analysis

To analyze study data we used inferential statistical methods. First, a probit model with sample selection was used to assess the relationship between receiving a rectal STI test and various sexual behaviors (receptive, insertive, versatile). Then, we used a probit model with sample selection to assess the relationship between receiving a rectal STI and sexual behavior disclosure/outness (non**-**specific disclosure, disclosure to health provider). We used STATA 13.0 [30]. All models included demographics, socioeconomic measures and sexual history as controls. Further information about statistical methods is in supplement. *P <* 0.05 was considered significant.

## Results

We approached 431 men intending to test for HIV and syphilis. After exclusion criteria and decision to participate, 301 men were enrolled and STI test uptake was 40%. Seven men chose to get both tests and were dropped from the analysis. As we are exploring whether sexual behavior is related to the choice of rectal over urethral testing, those who took both tests were not a focus of our analysis. Forty four % (50/114) chose the rectal gonorrhea and chlamydia test and 56% (64/114) picked the urethral gonorrhea and chlamydia test. Among the RCT participants, 35% (187/288) had disclosed sexual behavior to someone (non **-**specific disclosure) and 21% (59/288) of men had disclosed sexual behavior to their health provider. Five MSM were diagnosed with gonorrhea (urethral two, rectal three) and 19 with chlamydia (urethral six, rectal 13). We present descriptive statistics in Table 1.

**Table 1:** Participants characteristics

Variable	Mean (SD)
Age	28.10 (7.10)
Number of male partners last three months	2.30 (2.98)
	%
Gonorrhea test site	
rectal	43.9
urethal	56.1
	*n* = 114
Sexual behavior	
receptive	31.8
insertive	37.8
versatile	30.4
	*n* = 283
Yearly income, $	
< 2690.88	11.5
2690.88–5381.64	9.0
5381.64–8969.40	14.9
8969.40–14,351.04	26.4
> 14,351.04	38.2
	*n* = 288
Experienced STI symptoms	
no	11.2
yes	88.8
	*n* = 285
HIV test frequency	
< once every two years	16.9
once a year	23.0
once every six months	28.1
once every three months	26.3
monthly	5.8
	*n* = 278
Previous HIV test	
no	9.0
yes	91.0
	*n* = 288
Frequency of condomless anal intercourse last three months	
0% condom use	6.0
< 50% condom use	10.3
> 50% condom use	29.5
100% condom use	54.3
	*n* = 234
Out to someone (Non-specific disclosure)	
no	35.1
yes	64.9
	*n* = 288
Out to health provider (Disclosure to health provider)	
no	79.5
yes	20.5
	*n* = 288
Gonorrhea test result	
negative	98.3
positive	1.7
	*n* = 114
Chlamydia test result	
negative	93.7
positive	6.3
	*n* = 114

Using three separate models, we explored if MSM made a test choice in line with their indicated sexual behavior. Table 2 indicated that receptive sexual behavior was associated with 45.2% (95%CI = 33.8, 56.5) increased likelihood for selecting a rectal test.

**Table 2:** Multivariate analyses of MSM propensity to select the rectal test compared to the urethral test, in line with sexual behavior

Variable	Marginal Effects (95% CI)	*P*	Marginal Effects (95% CI)	*P*	Marginal Effects (95% CI)
Sexual behavior	Receptive		Insertive		Versatile
Dependent variable: rectal test					
Insertive	–	–	− 0.51 (− 0.59, − 0.44)	< .001	–
Receptive	0.45 (0.34, 0.57)	< .001	–	–	–
Versatile	–	–	–	–	0.006 (− 0.18, 0.19)
Age	0.006 (− 0.001, 0.013)	.12	0.004 (− 0.009, 0.018)	.52	0.003 (− 0.012, 0.019)
Income	0.012 (− 0.036, 0.060)	.64	0.050 (− 0.027, 0.128)	.2	0.05 (− 0.05, 0.14)
Number of male partners last three months	−0.022 (− 0.042, − 0.001)	.04	−0.019 (− 0.035, − 0.002)	.03	−0.01 (− 0.04, 0.01)
Frequency of condomless anal intercourse last three months	0.12 (− 0.031, 0.28)	.12	0.37 (0.17, 0.57)	< .001	0.26 (−0.13, 0.65)
Non-specific disclosure	−0.08 (− 0.19, 0.03)	.16	− 0.093 (− 0.34, 0.15)	.46	0.12 (− 0.1, 0.33)
Disclosure to health provider	0.04 (− 0.11, 0.18)	.6	−0.041 (− 0.29, 0.20)	.74	−0.046 (− 0.29, 0.20)
N	85		85		85
Predicted mean for receiving a rectal test	0.33		0.41		0.32

*Note:* Marginal effects of probit with sample selection (outcome equation results shown). Confidence interval (CI) estimated using jackknife with clustering by sites and within-site groups. Receptive: Compared to MSM not indicating the receptive role, MSM indicating the receptive role are more likely to select the rectal gonorrhea and chlamydia test, compared to the urethral test; Insertive: Compared to MSM not indicating the insertive role, MSM indicating the insertive role are less likely to select the rectal gonorrhea and chlamydia test, compared to the urethral test; Versatile: Compared to MSM not indicating the versatile role, MSM indicating the versatile role have no gonorrhea and chlamydia test preference

Insertive sexual behavior was related to 51.1% (95%CI = -58.7, − 43.5) decreased likelihood for selecting the rectal test. Finally, versatile sexual behavior was not significantly associated with selecting a rectal test, possibly indicating that versatile MSM have no preference for a rectal gonorrhea and chlamydia test.

**Fig. 1:**
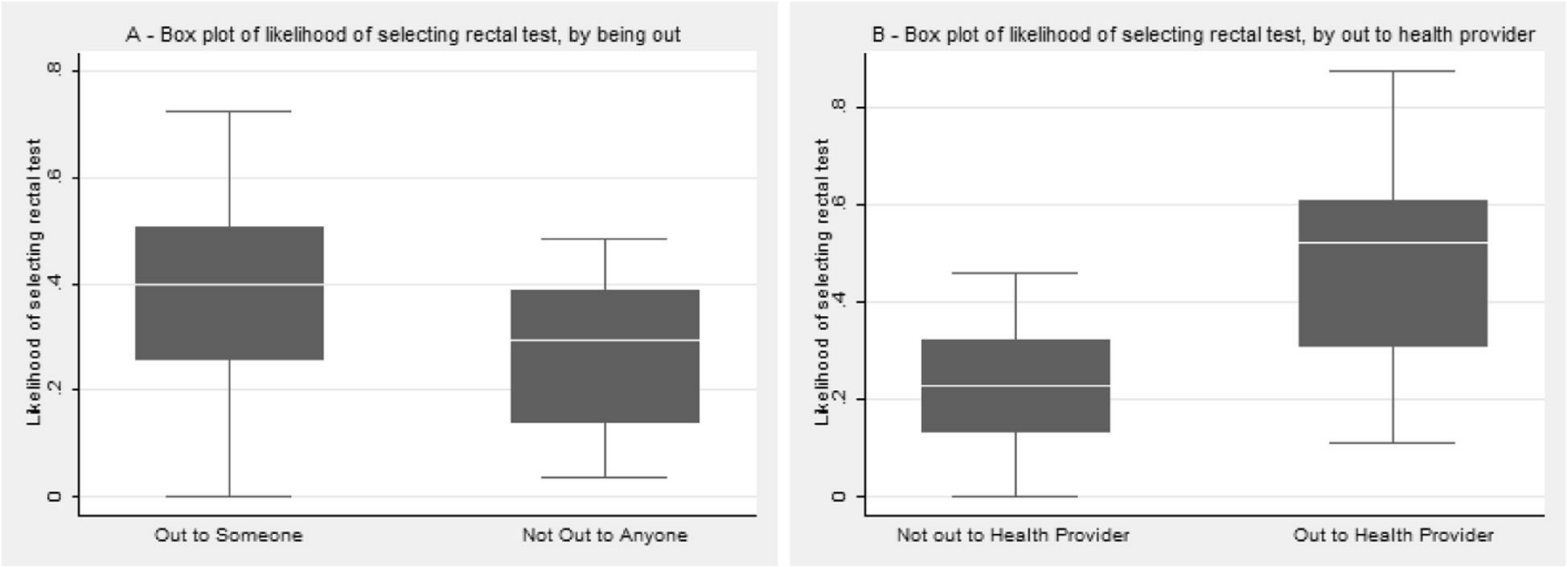
Probability of selecting a rectal gonorrhea and chlamydia test as a function of outness, for MSM preferring versatile sexual behavior. (Predicted probabilities shown, based on marginal effects estimated by respective models (accounting for selection) and the population sample)

We then explored disclosure and likelihood to select the rectal gonorrhea and chlamydia test. Table 3 indicated that there was no significant relationship between non**-**specific disclosure or disclosure to one’s health provider, and selecting a rectal gonorrhea and chlamydia test. Table 4 indicated that, for versatile MSM, non**-**specific disclosure was associated with a 26.8% (95%CI = 6.1, 47.5) increased likelihood of selecting the rectal gonorrhea and chlamydia test compared to the urethral test. We also found that for versatile MSM, disclosure to one’s health provider was associated with a 29.4% (95%CI = 6.3, 52.6) greater likelihood for selecting the rectal gonorrhea and chlamydia test, compared to the urethral test. These results were visualized in Fig. 1, focusing on the interaction effects between disclosure and versatile sexual behavior. While being versatile alone was not significantly associated with rectal test uptake, once non**-**specific disclosure or disclosure to health providers comes into the picture, the model suggested a large and significant increase in rectal test uptake. Note that this was a marginal effect, controlling for sociodemographics, sexual history and medical history relevant to STI testing.

**Table 3:** Multivariate analyses of MSM propensity to select the rectal test compared to the urethral test, in line with non-specific disclosure and disclosure to health provider

Variable	Marginal Effects (95% CI)	*P*	Marginal Effects (95% CI)	*P*
Type of disclosure	Non-specific disclosure		Disclosure to health provider	
Dependent variable: rectal test				
Insertive	–	–	–	–
Receptive	0.58 (0.5, 0.66)	< .001	0.58 (0.53, 0.64)	< .001
Versatile	0.26 (−0.23, 0.78)	0.29	0.26 (0.12, 0.41)	< .001
Age	0.01 (−0.01, 0.02)	.45	0.01 (−0.01, 0.02)	.35
Income	0.03 (−0.04, 0.1)	.47	0.02 (−0.04, 0.08)	.54
Number of male partners last three months	−0.02 (− 0.1, 0.05)	.51	− 0.03 (− 0.06, 0.002)	.07
Frequency of condomless anal intercourse last three months	0.23 (0.06, 0.4)	.01	0.23 (0.1, 0.36)	.001
Non-specific disclosure	−0.08 (− 0.32, 0.15)	.49	–	–
Disclosure to health provider	–	–	−0.04 (− 0.25, 0.17)	.72
N	85		85	
Predicted mean for receiving a rectal test	0.4		0.41	

*Note:* Marginal effects of probit with sample selection (outcome equation results shown). Confidence interval (CI) estimated using jackknife with clustering by sites and within-site groups. Non-specific disclosure: Compared to those not out to anyone, those out to someone are more likely to select the rectal gonorrhea and chlamydia test, compared to the urethral test; Disclosure to health provider: Compared to those not out to their health provider, those out to their health provider are more likely to select the rectal gonorrhea and chlamydia test, compared to the urethral test

**Table 4:** Multivariate analyses of versatile MSM propensity to select the rectal test compared to the urethral test, in line with non- specific disclosure and disclosure to health provider

Variable	Marginal Effects (95% CI)	*P*	Marginal Effects (95% CI)	*P*
Type of disclosure	Non-specific disclosure		Disclosure to health provider	
Dependent variable: rectal test				
Insertive	–	–	–	–
Receptive	0.61 (0.52, 0.70)	< .001	0.56 (0.46, 0.67)	< .001
Versatile	0.36 (0.23, 0.48)	< .001	0.15 (0.03, 0.26)	.01
Age	0.004 (−0.01, 0.01)	.46	0.005 (0.001, 0.01)	.01
Income	0.03 (−0.02, 0.08)	.24	0.02 (−0.02, 0.05)	.39
Number of male partners last three months	−0.03 (− 0.06, 0.003)	.08	− 0.03 (− 0.05, − 0.01)	.01
Frequency of condomless anal intercourse last three months	0.2 (0.09, 0.32)	.001	0.19 (0.05, 0.33)	.01
Non-specific disclosure	−0.2 (− 0.36, − 0.05)	.01	–	–
Versatile*non-specific disclosure	0.27 (0.06, 0.48)	.01	–	–
Disclosure to health provider	–	–	−0.16 (− 0.29, − 0.04)	.01
Versatile*disclosure to health provider	–	–	0.29 (0.06, 0.53)	.01
N	85		85	
Predicted mean for receiving a rectal test	0.41		0.39	

*Note:* Marginal effects of probit with sample selection (outcome equation results shown). Confidence interval (CI) estimated using jackknife with clustering by sites and within-site groups. Non-specific disclosure: Compared to versatile MSM not out to someone, versatile MSM who are out to someone (disclosed sexual identity) are more likely to select the rectal gonorrhea and chlamydia test, compared to the urethral test; Disclosure to health provider: Compared to versatile MSM not out to their health provider, versatile MSM out to their health provider are more likely to select the rectal gonorrhea and chlamydia test, compared to the urethral test

## Discussion

We first demonstrated that MSM selected tests in line with their preferred sexual behavior. We then indicated that versatile MSM out to a non**-**specific individual or one’s health provider (outside the study context) had increased likelihood for selecting the rectal gonorrhea and chlamydia test, compared to the urethral test. Our findings190 are in line with past research and reinforce the need to screen MSM for STIs through a full scope of transmission routes, ensuring no STIs are undiagnosed. We detailed how patient factors such as sexual behavior and outness may affect gonorrhea and chlamydia193 test provision in a clinical setting.

Many MSM in our sample with indications for rectal STI testing did not receive it. This is consistent with research in China and globally. A China based study found a higher prevalence of rectal chlamydia infection (24.4%) compared to urethral infection (5.3%) [31]. Similar findings were indicated in several other studies, where rectal prevalence of STIs was greater than the urethral prevalence [32–34]. Other global studies indicated similar findings. Among asymptomatic men screened for chlamydia, 9.8% were positive for rectal infection vs 2.3% for a urethral infection. However, the same study reported higher prevalence of urethral gonorrhea (5.0%) vs rectal gonorrhea (3.0%) [22]. Other studies indicated higher rates of rectal STI infections compared to urethral infections [35, 36]. Rectal STIs were associated with an increased risk for HIV seroconversion [37]. A retrospective MSM cohort study found that greater than two prior rectal gonorrhea or chlamydia infections were associated with eight times greater risk of HIV conversion [38]. Our findings indicated there could be a large number of missed infections and underestimation of STI prevalence. Undetected and consequently untreated cases may exacerbate the Chinese MSM STI epidemic [39]. We extended previous research suggesting the importance of rectal STI testing in MSM. MSM in marginalized contexts and resource limited settings may need to receive a combined rectal, urethral and pharyngeal gonorrhea and chlamydia test, as pharyngeal gonorrhea and chlamydia testing is also recommended for MSM [40]. However, when resources are scarce, as per our study, stigma free settings may allow for providing a single test most appropriate to sexual behavior.

Finally we found that MSM who had disclosed their sexual behavior to someone (non**-**specific disclosure) or their healthcare provider (outside the study context) were more likely to select rectal STI testing compared to urethral testing. Past China research indicated that larger disclosure networks were associated with greater propensity of HIV testing [18, 28]. Increased probability of never testing for HIV or syphilis was associated with non**-**disclosure to anyone or health professionals [41, 42]. The odds of disclosure to a healthcare professional was greater for MSM who had received an STI or HIV test [43]. In global literature, disclosure to healthcare providers was associated with HIV223 and STI testing among young MSM [44]. Closeted MSM were less likely to have tested for HIV compared to out MSM [27, 45]. Being completely out or even disclosure to a healthcare provider is clearly key to receiving STI and HIV testing, as Chinese MSM often express fear of being ostracized because of their sexual behavior, a common barrier preventing testing [46]. When MSM are given a choice between a rectal or urethral test, it is possible that patient factors affect test selection decision. We extend the literature to suggest that disclosure can improve testing outcomes.

### Limitations

This work has limitations. First, other unmeasured factors, such as knowledge levels about STIs and site of STI symptom (urethral or rectal), may have driven selection of the urethral gonorrhea and chlamydia test. We partially addressed this by controlling for previous HIV test, HIV test frequency, and possible STI symptoms in estimating the decision to test, but not the choice between the tests (since these measures are not site specific). We also conducted our analysis including education level as a control, but excluded it from the final analysis due to near colinearity with income. We did not consider how the psychological effects of testing would affect results. STI testing can be viewed as a form of commitment in a relationship [47] or cause significant distress [48].

Further work can model this through a survey item or qualitative techniques. Second, the gonorrhea and chlamydia test RCT was conducted at sites catered to MSM STI testing. Such site selection may have limited analysis to MSM connected with community based organizations and already interested in HIV testing [49]. Despite limited generalizability to hospitals and other provider settings, our results remain relevant since specialized community MSM clinics remain major providers of testing in China [50] and globally [51] where patient factors drive health outcomes. As participants would have to pay an247 additional amount to take both tests, it could be that some selected a single test due to lack of funds. We utilized income as a control to account for this concern. Due to resource limitations, we were unable to offer rectal and urethral testing to all participants and then determine the number of mismatches between a positive test at a particular site and sexual behavior (e.g. MSM reporting insertive sexual behavior but with a positive2 rectal test). Future research will incorporate such a study design.

## Conclusion

Greater efforts are needed to ensure that patient factors do not adversely affect MSM testing outcomes. Sexual behavior and outness may affect gonorrhea and chlamydiatesting provision. Apart from clinicians, community based efforts may reduce stigma based barriers to testing.

## Supplementary information

**Additional file 1.** Supplement [52–54].

**Additional file 2 Table S1.** Outness, Sexual Behavior and Gonorrhea and Chlamydia Test Choice Among Chinese MSM.

**Additional file 3.** Supplement patient survey.

## Data Availability

The datasets generated and/or analyzed for this study are not publicly available dueto privacy issues but are available from the corresponding author on reasonable request.

## Abbreviations

STI: Sexually transmitted infection
MSM: Men who have sex with men
RCT: Randomized controlled trial
