# Supplementary material for: Lack of sexual behavior disclosure may distort STI testing outcomes
Source: BMC Public Health. 2020 May 4;20:616. doi: 10.1186/s12889-020-08768-5 (PMC7197169; doi:10.1186/s12889-020-08768-5)
Supplement: Supplementary file 1 — Additional file 1. Supplement [52–54]. [file 12889_2020_8768_MOESM1_ESM.docx]

**Supplement**

**Study design**

The parent RCT was designed to evaluate the comparative effectiveness of Pay-it-Forward and Pay-what-you-Want against the standard of care for increasing gonorrhea and chlamydia test uptake in MSM. Pay-it-forward (PIF) consists of telling participants that their test was paid for by another MSM and asking the participant how much they would like to contribute to the next participant.[52] Pay-what-you-Want (PW) is a pricing strategy where consumers select a desired amount for a product or service.[53] In a solely monetary sense, PW is like PIF pricing where consumers select the price for a good or service. However, PW and PIF vary socially. In PW, the consumer pays for themselves, while in PIF, the consumer pays for someone else. Comparing PW and PIF can provide insight on whether participants are engaging in testing solely because it is free or if there are community-based effects of altruism involved.

Participants were randomly assigned into clusters within the study arms. A cluster is a group of ten eligible men who arrived one after another at the study sites and decided to participate. Cluster randomization was utilized to minimize intervention contamination to account for MSM who turned up in pairs and to simplify processes undertaken by site staff. MSM in the same cluster were collectively assigned to the same study arm. Similarly, those who arrived with partners were placed in the same study arm. RCT randomization sequence was designed through STATA 15. For the PIF arm, 9 participants were told the experiment was for promoting gonorrhea and chlamydia test uptake and that the standard price of a gonorrhea and chlamydia test was 150RMB (US$22). They were offered a free test and told it was paid for by another MSM. In the PW introduction, MSM were told the standard gonorrhea and chlamydia test price was 150RMB (US$22). MSM were told that they could first receive a free gonorrhea and chlamydia test and then decide the amount to pay. Participants were told that payment and receiving a gonorrhea and chlamydia test was voluntary and the payment amount was up to the participant. MSM assigned to the SOC arm received the same gonorrhea and chlamydia test information through a pamphlet and no further details. Those in the SOC arm were told that the standard price of the gonorrhea and chlamydia test was 150RMB (US$22). 101 were allocated to the PIF arm, 100 to the PW arm and 100 to the SOC arm across the three testing sites. Within the RCT, gonorrhea and chlamydia test uptake was 56%, 46%, and 18% respectively.

# Model specification

A significant concern in our analysis was the possibility of selection bias. Those who did not want the gonorrhea and chlamydia test may be different from men who received the gonorrhea and chlamydia test. This issue is prevalent in most testing environments.

Selection of gonorrhea and chlamydia test would lead to invalid tests of our research questions if the factors responsible for the decision of taking the test are also related to our main variables of interest. We overcame this problem by using random assignment as an instrument. We applied a probit model with sample selection (an extension of the Heckman selection model for outcomes that are binary rather than continuous) [54].

We model the decision to take the test and the subsequent test choice. Test uptake (selection stage) was operationalized as a dichotomous variable indicating whether men had selected the gonorrhea and chlamydia test. In the outcome stage, our variable of interest was choice of rectal gonorrhea and chlamydia test. Standard errors were calculated with a jackknife estimator, using 30 jackknife samples that accounted for the study design. The first-stage model applies a probit model to represent a measure of the propensity of a participant to choose a gonorrhea and chlamydia test on the basis of factors believed to be related to the decision to test. The first model’s assessment of the probability of the gonorrhea and chlamydia test choice is then applied in the second520 stage to adjust the estimates produced from a probit model to account for the effect of selection bias. To effectively use this approach, we had to develop an understanding not only of factors that influenced selection of the rectal gonorrhea and chlamydia test but also of factors that affected the decision to engage in the gonorrhea and chlamydia test uptake. Formally, we can write the two models as follows:


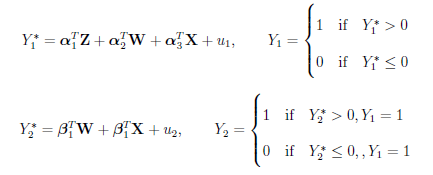


where Y_1_^*^ represents the decision to test and Y_2_^*^ represents the decision to select the rectal gonorrhea and chlamydia test as unobserved latent variables. **Z** is the set of instruments used to adjust for selection, **W** is the set of variables of interest for which we wish to infer the effect on the outcome *Y*_2_, and **X** is the set of controls for the outcome model. **Z** were included in the selection stage but not the outcome stage: Whether participant had experienced STI symptoms prior to the gonorrhea and chlamydia test, Previous HIV test, HIV test frequency, Site, Arm. These variables likely influenced test uptake but not gonorrhea and chlamydia test choice and were generally not associated with gonorrhea and chlamydia test choice. **W**: Insertive, Receptive, Versatile, Out to anyone, Out to health provider. Inclusion of W was dependent on the hypothesis of interest. **X**: Age, Income, Number of male partners in last three months, Frequency of condomless anal intercourse.

Given the relatively small number of participants both versatile and out, we were limited in the number of controls to include. We thus did not include controls co linear with reported variables (e.g. marital status, education). Regarding the STI symptoms variables, participants were not asked where on the body symptoms were observed, just whether they had symptoms. Thus, the symptoms variable may affect test uptake but not rectal gonorrhea and chlamydia test choice, perhaps indicative of a strong instrument. The Arm variable varies the attractiveness of testing, but does not affect rectal gonorrhea and chlamydia test choice, perhaps indicating its strength as an instrument.
