# Supplementary material for: Lack of sexual behavior disclosure may distort STI testing outcomes
Source: BMC Public Health. 2020 May 4;20:616. doi: 10.1186/s12889-020-08768-5 (PMC7197169; doi:10.1186/s12889-020-08768-5)
Supplement: Supplementary file 2 — Additional file 2 Table S1. Outness, Sexual Behavior and Gonorrhea and Chlamydia Test Choice Among Chinese MSM. [file 12889_2020_8768_MOESM2_ESM.docx]

**Table 1S** Outness, Sexual Behavior and Gonorrhea and Chlamydia Test Choice Among Chinese MSM

| Variable | Coefficients (95% CI) | *P* | Coefficients (95% CI) | *P* | Coefficients (95% CI) | *P* | Coefficients (95% CI) | *P* | Coefficients (95% CI) | *P* | Coefficients (95% CI) | *P* | Coefficients (95% CI) | *P* |
| --- | --- | --- | --- | --- | --- | --- | --- | --- | --- | --- | --- | --- | --- | --- |
|  | Receptive |  | Insertive |  | Versatile |  | Non-specific disclosure |  | Disclosure to health provider |  | Versatile MSM-Non-specific disclosure |  | Versatile MSM-Disclosure to health provider |  |
| Outcome stage. Dependent variable: rectal test  Insertive | - | - | -2.12 (-3.58, -0.82) | .003 | - | - | - | - | - | - | - | - | - | - |
| Receptive | 1.98 (0.94, 3.03) | .001 | - | - | - | - | 3.04 (-2.79, 8.87) | .29 | 3.13 (1.7, 4.56) | < .001 | 3.3 (2.11, 4.48) | < .001 | 3.24 (1.64, 4.85) | .04 |
| Versatile | - | - | - | - | 0.02 (-0.55, 0.6) | .95 | 1.38 (0.18, 2.58) | .03 | 1.41 (0.24, 2.57) | .02 | 1.92 (1.13, 2.71) | < .001 | 0.86 (0.02, 1.69) | < .001 |
| Age | 0.03 (-0.01, 0.06) | .09 | 0.02 (-0.04, 0.08) | .55 | 0.01 (-0.04, 0.06) | .71 | 0.03 (-0.04, 0.09) | .42 | 0.031 (-0.04, 0.1) | .38 | 0.02 (-0.04, 0.08) | .47 | 0.03 (0.01, 0.06) | .02 |
| Income | 0.05 (-0.17, 0.28) | .66 | 0.22 (-0.2, 0.64) | .3 | 0.13 (-0.15, 0.42) | .34 | 0.13 (-0.4, 0.67) | .61 | 0.11 (-0.26, 0.47) | .56 | 0.26 (-0.13, 0.46) | .34 | 0.09 (-0.14, 0.32) | .42 |
| Number of male partners last three months | -0.1 (-0.2, 0.01) | .07 | -0.08 (-0.16, -0.002) | .04 | -0.04 (-0.11, 0.03) | .27 | -0.13 (-0.75, 0.5) | .69 | -0.15 (-0.33, 0.03) | .1 | -0.14 (-0.32, 0.04) | .12 | -0.17 (-0.36, 0.01) | .06 |
| Frequency of condomless anal intercourse last three months | 0.56 (-0.22, 1.31) | .15 | 1.6 (0.38, 2.82) | .01 | 0.76 (-0.29, 1.81) | .15 | 1.2 (-0.89, 3.33) | .25 | 1.22 (0.38, 2.06) | .01 | 1.09 (0.35, 1.83) | .01 | 1.08 (-0.1, 2.07) | .03 |
| Non-specific disclosure | -0.34 (-0.88, 0.19) | .2 | -0.4 (-1.65, 0.85) | .52 | 0.35 (-0.28, 0.98) | .27 | -0.44 (-1.54, 0.66) | .42 | - | - | -1.09 (-1.96, -0.22) | .02 | - | - |
| Versatile*non-specific disclosure | - | - | - | - | - | - | - | - | - | - | 1.45 (0.29, 2.61) | .02 | - | - |
| Disclosure to health provider | 0.17 (-0.52, 0.89) | .62 | -0.18 (-1.32, 0.97) | .76 | 0.13 (-0.62, 0.89) | .72 | - | - | -0.21 (-1.45, 1.03) | .73 | - | - | -0.95 (-1.67, -0.23) | .01 |
| Versatile*disclosure to health provider | - | - | - | - | - | - | - | - | - | - | - | - | 1.7 (0.37, 3.04) | .02 |
| Selection stage. Dependent variable: test uptake Insertive | - | - | -0.08 (-0.44, 0.28) | .67 | - | - | - | - | - | - | - | - | - | - |
| Receptive | 0.03 (-0.31, 0.38) | .85 | - | - | - | - | 0.06 (-0.36, 0.49) | .76 | 0.06 (-0.33, 0.46) | .76 | 0.057 (-0.35, 0.46) | .77 | 0.05 (-0.32, 0.43) | .77 |
| Versatile | - | - | - | - | 0.56 (-0.15, 1.27) | .78 | 0.09 (-0.33, 0.52) | .65 | 0.07 (-0.36, 0.5) | .73 | 0.3 (-0.34, 0.94) | .34 | -0.08 (-0.56, 0.41) | .75 |
| Age | 0.03 (0.002, 0.06) | .04 | 0.03 (-0.002, 0.07) | .06 | 0.03 (-0.003, 0.07) | .07 | 0.03 (-0.001, 0.07) | .06 | 0.03 (-0.01, 0.07) | .1 | 0.03 (-0.003, 0.06) | .07 | 0.03 (-0.01, 0.06) | .1 |
| Income | -0.16 (-0.32, 0.003) | .05 | -0.16 (-0.34, 0.02) | .08 | -0.16 (-0.34, 0.02) | .08 | -0.16 (-0.33, 0.02) | .08 | -0.15 (-0.32, 0.03) | .1 | -0.14 (-0.32, 0.03) | .1 | -0.14 (-0.31, 0.02) | .09 |
| Number of male partners last three months | 0.05 (-0.07, 0.17) | .38 | 0.06 (-0.07, 0.18) | .36 | 0.05 (-0.07, 0.17) | .36 | 0.06 (-0.07, 0.18) | .35 | 0.06 (-0.07, 0.18) | .35 | 0.06 (-0.07, 0.18) | .37 | 0.06 (-0.06, 0.17) | .34 |
| Frequency of condomless anal intercourse last three months | -0.41 (-1.56, 0.73) | .46 | -0.43 (-1.57, 0.7) | .44 | -0.42 (-1.53, 0.7) | .45 | -0.41 (-1.56, 0.73) | .47 | -0.42 (-1.53, 0.68) | .44 | -0.41 (-1.7, 0.89) | .53 | -0.41 (-1.62, 0.81) | .5 |
| HIV test frequency | -0.01 (-0.21, 0.19) | .94 | -0.004 (-0.23, 0.22) | .97 | -0.003 (-0.24, 0.23) | .98 | -0.000 (-0.22, 0.22) | 1 | -0.003 (-0.22, 0.21) | .98 | -0.009 (-0.22, 0.21) | .93 | 0.003 (-0.21, 0.22) | 1 |
| Previous HIV test | 1.01 (0.37, 1.65) | .003 | 1.03 (0.38, 1.67) | .003 | 1.04 (0.41, 1.68) | .002 | 1.04 (0.38, 1.7) | .003 | 0.97 (0.36, 1.59) | .003 | 1.12 (0.5, 1.73) | .001 | 0.96 (0.33, 1.59) | .004 |
| Possible STI Symptoms | 0.60 (-0.03, 1.23) | .06 | 0.6 (-0.09, 1.28) | .09 | 0.56 (-0.15, 1.27) | .12 | 0.62 (-0.05, 1.28) | .07 | 0.62 (-0.07, 1.3) | .07 | 0.63 (-0.06, 1.32) | .07 | 0.63 (-0.04, 1.3) | .07 |
| Non-specific disclosure | 0.22 (0.19, 0.63) | .28 | - | - | 0.24 (-0.23, 0.7) | .31 | 0.27 (-0.19, 0.73) | .24 | - | - | 0.09 (-0.52, 0.7) | .76 | - | - |
| Versatile*non-specific disclosure | - | - | - | - | - | - | - | - | - | - | 0.62 (-0.67, 1.9) | .33 | - | - |
| Disclosure to health provider | 0.19 (-0.28, 0.66) | .42 | 0.11 (-0.45, 0.66) | .7 | 0.11 (-0.43, 0.64) | .68 | - | - | 0.21 (-0.33, 0.75) | .43 | - | - | 0.06 (-0.56, 0.68) | .85 |
| Versatile*disclosure to health provider | - | - | - | - | - | - | - | - | - | - | - | - | 0.61 (-1.03, 2.25) | .45 |
| Site  -site 2 | -0.61 (-1.18, -0.03) | .04 | -0.69 (-1.93, 0.55) | .26 | -0.7 (-1.93, 0.53) | .25 | -0.68 (-1.88, 0.53) | .26 | -0.66 (-1.85, 0.54) | .27 | -0.68 (-1.87, 0.51) | .25 | -0.66 (-1.56, 0.24) | .14 |
| -site 3 | -0.18 (-0.59, 0.24) | .39 | -0.3 (-1.45, 0.84) | .6 | -0.29 (-1.39, 0.82) | .6 | -0.28 (-1.41, 0.85) | .61 | -0.28 (-1.4, 0.84) | .61 | -0.29 (-1.4, 0.82) | .6 | -0.26 (-1.07, 0.56) | .53 |
| Arm  -pay-it-forward | 1.36 (0.71, 2.01) | < .001 | 1.31 (0.57, 2.04) | .001 | 1.29 (0.52, 2.05) | .25 | 1.34 (0.61, 2.06) | .001 | 1.31 (0.59, 2.04) | .001 | 1.38 (0.66, 2.09) | .001 | 1.3 (0.64, 1.97) | < .001 |
| -pay-what you-want | 0.99 (0.32, 1.68) | .01 | 0.97 (0.18, 1.78) | .02 | 1.02 (0.27, 1.77) | .01 | 0.97 (0.2, 1.75) | .02 | 0.96 (0.17, 1.76) | .02 | 1 (0.2, 1.8) | .02 | 0.95 (0.17, 1.737) | .02 |
| N | 85 |  | 85 |  | 85 |  | 85 |  | 85 |  | 85 |  | 85 |  |

*Note:* Coefficients of probit with sample selection. Confidence interval (CI) estimated using jackknife with clustering by sites and within-site groups. Receptive: Compared to MSM not indicating the receptive role, MSM indicating the receptive role are more likely to select the rectal gonorrhea and chlamydia test, compared to the urethral test; Insertive: Compared to MSM not indicating the insertive role, MSM indicating the insertive role are less likely to select the rectal gonorrhea and chlamydia test, compared to the urethral test; Versatile: Compared to MSM not indicating the versatile role, MSM indicating the versatile role have no gonorrhea and chlamydia test preference; Non-specific disclosure: Compared to those not out to anyone, those out to someone are more likely to select the rectal gonorrhea and chlamydia test, compared to the urethral test; Disclosure to health provider: Compared to those not out to their health provider, those out to their health provider are more likely to select the rectal gonorrhea and chlamydia test, compared to the urethral test; Versatile MSM-Non-specific disclosure: Compared to versatile MSM not out to someone, versatile MSM who are out to someone (disclosed sexual identity) are more likely to select the rectal gonorrhea and chlamydia test, compared to the urethral test; Versatile MSM-Disclosure to health provider: Compared to versatile MSM not out to their health provider, versatile MSM out to their health provider are more likely to select the rectal gonorrhea and chlamydia test, compared to the urethral test.
