# Supplementary material for: Lack of sexual behavior disclosure may distort STI testing outcomes
Source: BMC Public Health. 2020 May 4;20:616. doi: 10.1186/s12889-020-08768-5 (PMC7197169; doi:10.1186/s12889-020-08768-5)
Supplement: Supplementary file 3 — Additional file 3. Supplement patient survey. [file 12889_2020_8768_MOESM3_ESM.docx]

**Supplemental Patient Survey for Pay- It-Forward Project** Date- Number:

1.Anus 2. Urethra 3. Throat

S3. Location:

1. Yes 2. No

S2. Participation in PIF:

**PRE-PARTICIPATION QUESTIONS**

S1. Here for testing: 1. Alone 2. With friend/partner

**SOCIODEMOGRAPHICS**

A1. Age: years A2. Nationality

1. Han Chinese 2. Other A3. Current marital status:

1. Never married 2. Married

3. Widowed, separated or divorced A4. Highest level of completed education:

1. Elementary 2. Middle

3. High school 4. Vocational college

5. Bachelor or above

B5. In the past 3 months, have you had condomless oral sex?

1. Yes 2. No

B6. In the past, have you told anyone about your sexuality or sexual history with men? (Select all that apply)

1. Yes, my long-term female partner/wife
2. Yes, my family members
3. Yes, my friends
4. Yes, my healthcare providers
5. No one

**CLINICAL INFORMATION**

C1. Do you have any symptoms that you are worried may be due to an STI?

1. Yes. Symptoms: 2. No

C2. Has anyone (a friend, family member, sexual partner, health professional) suggested that you get tested for gonorrhea or chlamydia?

1. I don’t know anything about gonorrhea or chlamydia
2. I don’t want to know if I have gonorrhea or chlamydia
3. I don’t need to get tested
4. Too much of a hassle
5. Too expensive
6. I am worried about confidentiality
7. I am afraid of pain/ discomfort
8. I am embarrassed to get a sample taken
9. I am embarrassed to get tested in front of my friend/partner
10. I am afraid that my results will be positive
11. Other

**PIF PARTICIPATION**

P1. Did you get free chlamydia and gonorrhea tests today through PIF?

1. Yes (Go to P2) 2. No (Go to P3)

P2. If Yes, how much did you pay it forward?

A5. Hukou residency:

1. Urban 2. Rural

A6. Individual Income (RMB/month)

1. Yes. Relationship with you: C3. Have you ever tested for HIV in the past?

1. Yes: date of last test
2. No
3. No

P3. If No, why NOT?

- 1. I did not want to get gonorrhea or chlamydia testing today
  2. I did not want to participate in PIF

1. 0-1000 2. 1000-5000

C4. Have you ever tested for gonorrhea in the past?

P4. What do you believe are the main benefits to

3. 5000-10,000 4. > 10,000 RMB/month

1. Yes: date of last test
2. No

participating in the PIF program? (select all that apply)

**SEXUAL HISTORY**

C5. Have you ever tested for chlamydia in the past?

1. Discounted GC/CT test

B1. Have you ever had anal sex with a man?

1. Yes: date of last test
2. No
3. I can learn about my own STI status
4. More MSM can get tested
   1. Yes 2. No

B2. If yes, what is your role during anal sex?

1. Mostly receptive (bottom)
2. Half and half (versatile)
3. Mostly insertive (top)

B3. In the past 3 months, how many sex partners have you had? (Number)

male partners female partners

B4. In the past 3 months, have you had condomless anal sex?

1. Yes 2. No

C6. Did you get tested for gonorrhea and chlamydia today?

1. Yes (Go to C7) 2. No (Go to C8)

C7. If you got tested for gonorrhea and chlamydia today, why is the MAIN reason you got tested? (**Choose ONE**)

1. Recent symptoms
2. Recent high risk sexual behavior
3. A friend told me to get tested
4. A doctor or nurse told me to get tested
5. The Pay-it-forward project
6. Other

C8. If you did NOT get tested for gonorrhea and chlamydia today, why NOT? (select all that apply)

1. Someone has helped me, and I can help someone else
2. Other

P5. What do you believe are the main barriers to participating in the PIF program? (select all that apply)

1. Difficult to understand
2. Too much trouble
3. I prefer to pay for my own testing
4. I don’t want to donate money to others’ testing
5. I am anxious that my donation is not enough
6. Other
7. I don’t think there are any barriers

**Test results** HIV: Syphilis: CT: GC:
